# Supplementary material for: Interleukin-2-mediated NF-κB-dependent mRNA splicing modulates interferon gamma protein production
Source: EMBO Rep. 2024 Nov 22;26(1):16–35. doi: 10.1038/s44319-024-00324-1 (PMC11724048; doi:10.1038/s44319-024-00324-1)
Supplement: Supplementary file 3 — Source data Fig. 2 [file 44319_2024_324_MOESM3_ESM.zip › 2B/2100 expert_DNA 7500_DE13805338_2021-10-06_16-45-39.pdf]

Assay Class: DNA 7500
Data Path: C:\...ety\2100 expert_DNA 7500_DE13805338_2021-10-06_16-45-39.xad

Created: 10/6/2021 4:45:38 PM
Modified: 10/6/2021 5:20:32 PM

Electrophoresis File Run Summary

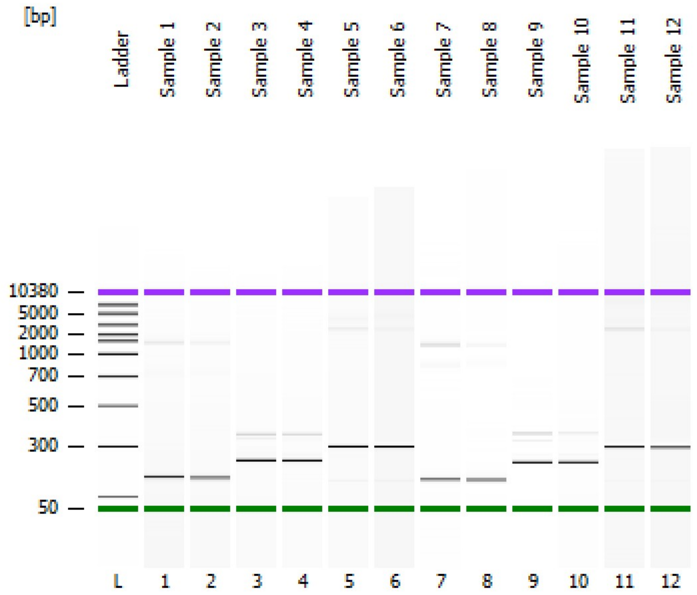

<u>Instrument Information:</u>

Instrument Name: DE13805338 Firmware: C.01.069
Serial#: DE13805338 Type: G2939A

<u>Assay Information:</u>

Assay Origin Path: C:\Program Files\Agilent\2100 bioanalyzer\2100 expert\assays\dsDNA\DNA 7500 Series II.xsy

Assay Class: DNA 7500

Version: 2.3

Assay Comments: DNA Analysis 100 -7500 bp

© Copyright 2003-2009 Agilent Technologies, Inc.

<u>Chip Information:</u>

Chip Lot #:

Reagent Kit Lot #:

Chip Comments:

Sample 1

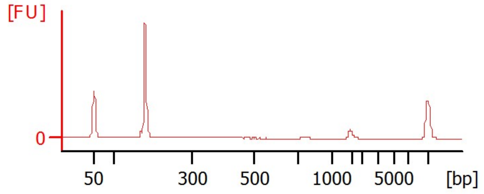

Sample 2

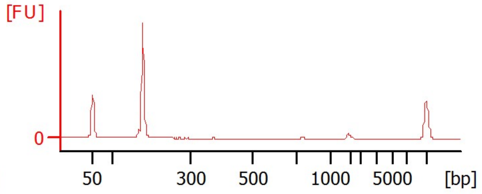

Sample 3

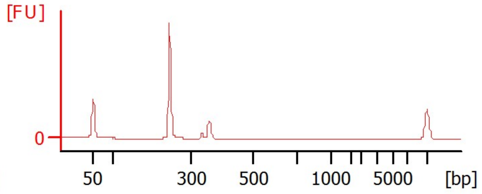

Sample 4

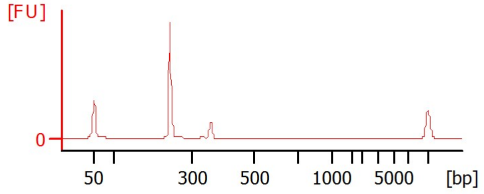

Sample 5

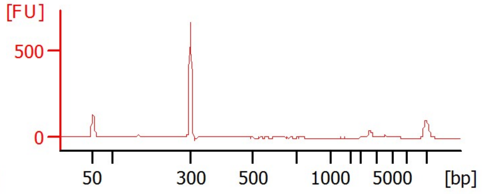

Sample 6

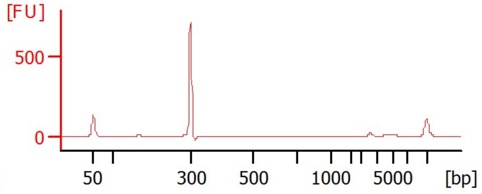

Sample 7

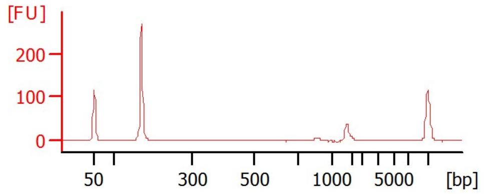

Sample 8

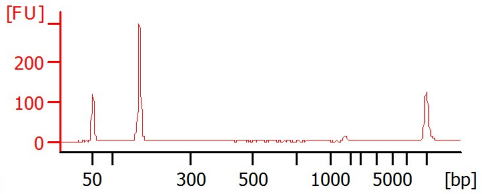

Sample 9

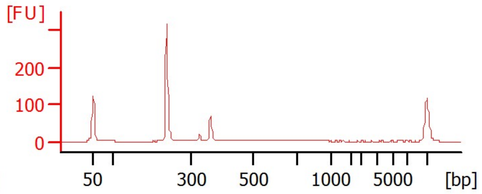

Sample 10

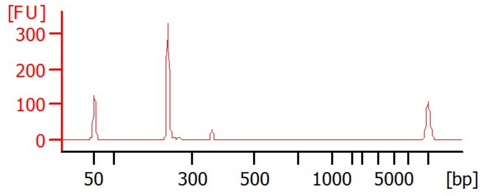

Sample 11

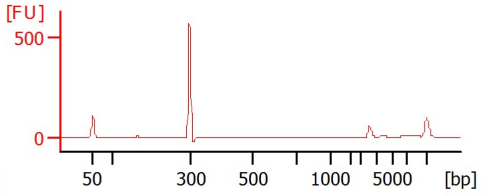

Sample 12

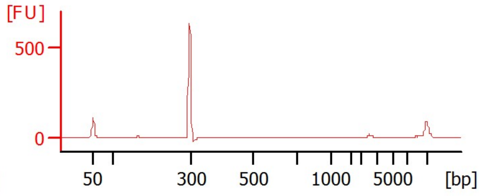

Assay Class: DNA 7500
Data Path: C:\...ety\2100 expert_DNA 7500_DE13805338_2021-10-06_16-45-39.xad
Created: 10/6/2021 4:45:38 PM
Modified: 10/6/2021 5:20:32 PM

Electrophoresis File Run Summary (Chip Summary)

Sample Name	Sample Comment	Rest. Digest	Status	Observation	Result Label	Result Color
Sample 1		☐	✔			
Sample 2		☐	✔			
Sample 3		☐	✔			
Sample 4		☐	✔			
Sample 5		☐	✔			
Sample 6		☐	✔			
Sample 7		☐	✔			
Sample 8		☐	✔			
Sample 9		☐	✔			
Sample 10		☐	✔			
Sample 11		☐	✔			
Sample 12		☐	✔			
Ladder		☐	✔			

Chip Lot # **Reagent Kit Lot #**

Chip Comments :

© Copyright 2003 - 2009 Agilent Technologies, Inc.

Assay Class: DNA 7500
Data Path: C:\...ety\2100 expert_DNA 7500_DE13805338_2021-10-06_16-45-39.xad
Created: 10/6/2021 4:45:38 PM
Modified: 10/6/2021 5:20:32 PM

Electrophoresis Assay Details

General Analysis Settings

Number of Available Sample and Ladder Wells (Max.) : 13
Minimum Visible Range [s] : 20
Maximum Visible Range [s] : 94
Start Analysis Time Range [s] : 20
End Analysis Time Range [s] : 93.95
Ladder Concentration [ng/µl] : 40
Uses Standard Area for Ladder Fragments
Lower Marker Concentration [ng/µl] : 8.3
Upper Marker Concentration [ng/µl] : 4.2
Used Upper Marker for Quantitation
Standard Curve Fit is Point to Point
Show Data Aligned to Lower and Upper Marker

Integrator Settings

Integration Start Time [s] : 20
Integration End Time [s] : 93.95
Slope Threshold : 0.8
Height Threshold [FU] : 20
Area Threshold : 0.1
Width Threshold [s] : 0.5
Baseline Plateau [s] : 0.5

Filter Settings

Filter Width [s] : 0.5
Polynomial Order : 4

Ladder

Ladder Peak	Size	Area
1	50	120
2	100	47
3	300	63
4	500	81
5	700	85
6	1000	93
7	1500	101
8	2000	101
9	3000	106
10	5000	108
11	7000	109
12	10380	107

 © Copyright 2003 - 2009 Agilent Technologies, Inc.

Assay Class: DNA 7500
Data Path: C:\...ety\2100 expert_DNA 7500_DE13805338_2021-10-06_16-45-39.xad

Created: 10/6/2021 4:45:38 PM
Modified: 10/6/2021 5:20:32 PM

Electropherogram Summary

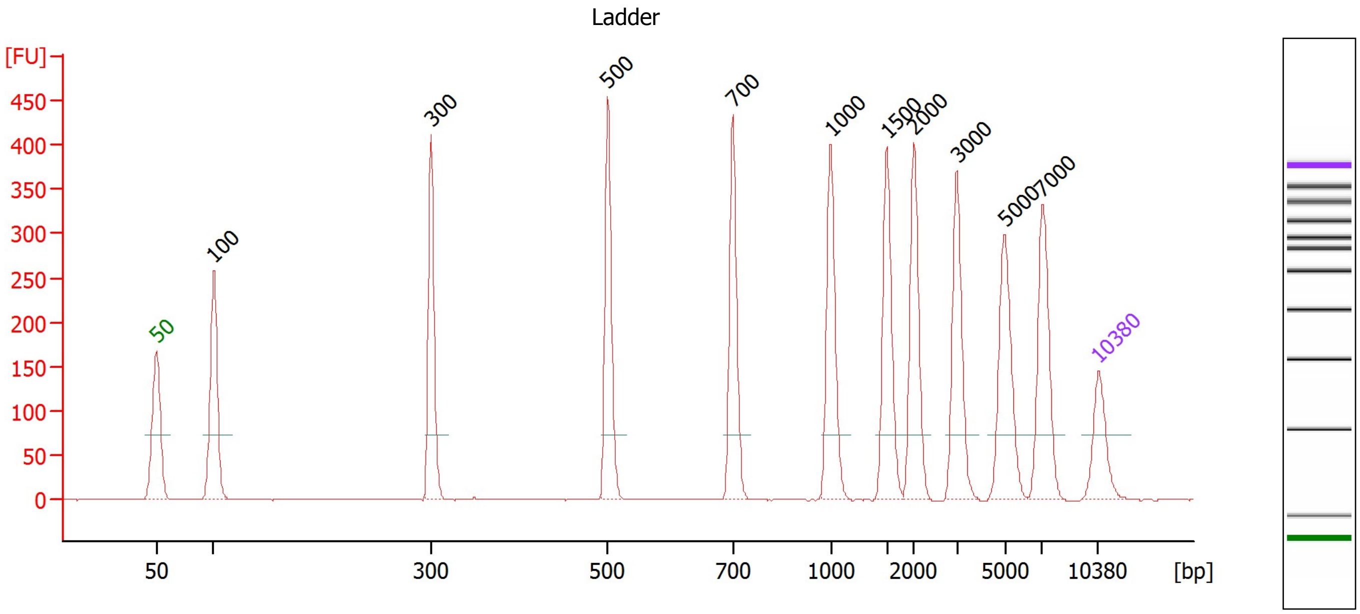

Peak table for Ladder

Peak		Size [bp]	Conc. [ng/µl]	Molarity [nmol/l]	Observations
1		50	8.30	251.5	Lower Marker
2		100	4.00	60.6	Ladder Peak
3		300	4.00	20.2	Ladder Peak
4		500	4.00	12.1	Ladder Peak
5		700	4.00	8.7	Ladder Peak
6		1,000	4.00	6.1	Ladder Peak
7		1,500	4.00	4.0	Ladder Peak
8		2,000	4.00	3.0	Ladder Peak
9		3,000	4.00	2.0	Ladder Peak
10		5,000	4.00	1.2	Ladder Peak
11		7,000	4.00	0.9	Ladder Peak
12		10,380	4.20	0.6	Upper Marker

 © Copyright 2003 - 2009 Agilent Technologies, Inc.

Assay Class: DNA 7500 Created: 10/6/2021 4:45:38 PM
Data Path: C:\...ety\2100 expert_DNA 7500_DE13805338_2021-10-06_16-45-39.xad Modified: 10/6/2021 5:20:32 PM

Electropherogram Summary Continued ...

Sample 1

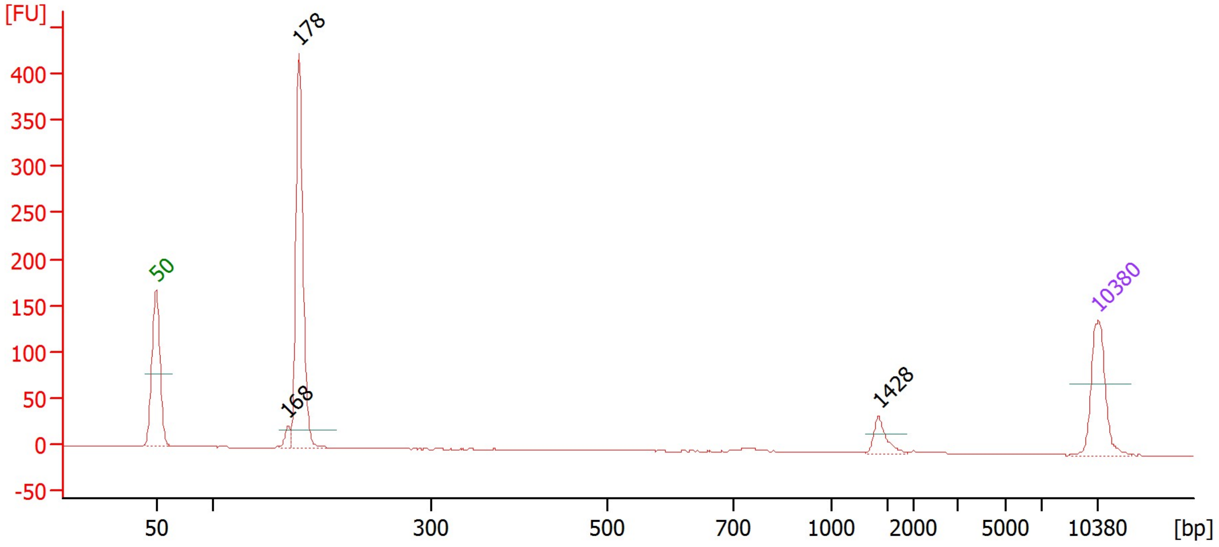

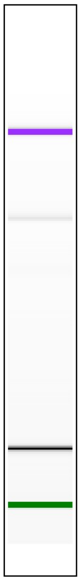

Overall Results for sample 1 : Sample 1

Number of peaks found: 3

Peak table for sample 1 : Sample 1

Peak		Size [bp]	Conc. [ng/µl]	Molarity [nmol/l]	Observations
1	◀	50	8.30	251.5	Lower Marker
2		168	0.47	4.2	
3		178	11.81	100.4	
4		1,428	1.02	1.1	
5	▶	10,380	4.20	0.6	Upper Marker

 © Copyright 2003 - 2009 Agilent Technologies, Inc.

Assay Class: DNA 7500 Created: 10/6/2021 4:45:38 PM
Data Path: C:\...ety\2100 expert_DNA 7500_DE13805338_2021-10-06_16-45-39.xad Modified: 10/6/2021 5:20:32 PM

Electropherogram Summary Continued ...

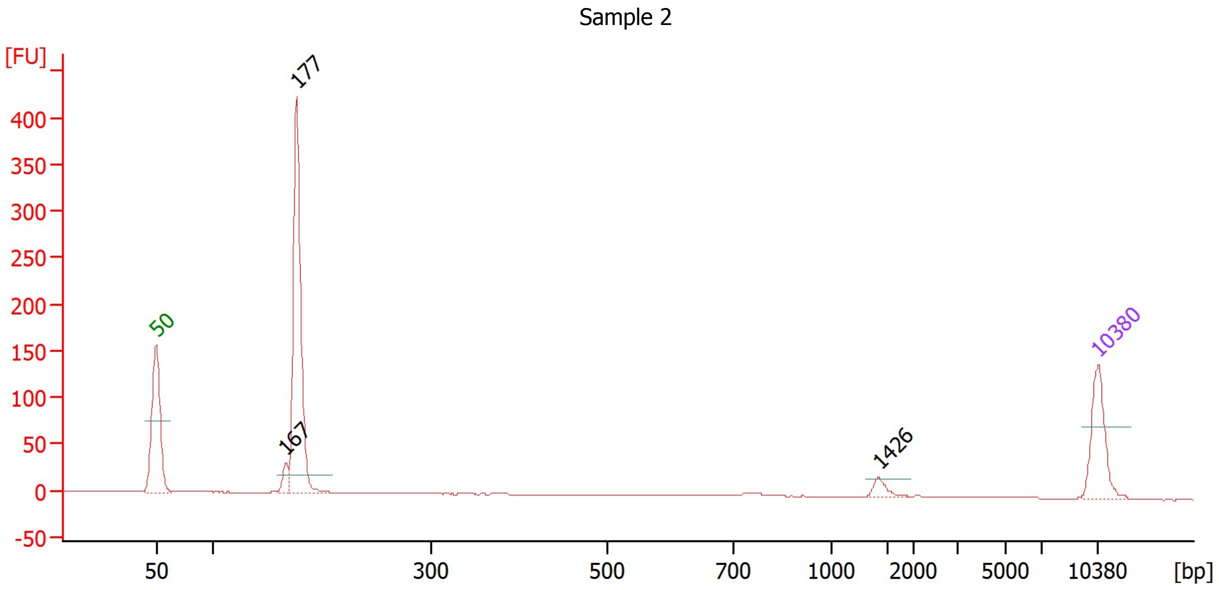

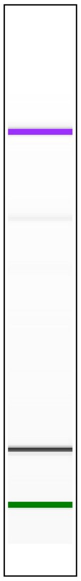

Overall Results for sample 2 : **Sample 2**

Number of peaks found: 3

Peak table for sample 2 : **Sample 2**

Peak		Size [bp]	Conc. [ng/µl]	Molarity [nmol/l]	Observations
1	◀	50	8.30	251.5	Lower Marker
2		167	0.74	6.8	
3		177	13.07	112.2	
4		1,426	0.67	0.7	
5	▶	10,380	4.20	0.6	Upper Marker

Assay Class: DNA 7500
Data Path: C:\...ety\2100 expert_DNA 7500_DE13805338_2021-10-06_16-45-39.xad
Created: 10/6/2021 4:45:38 PM
Modified: 10/6/2021 5:20:32 PM

Electropherogram Summary Continued ...

Sample 3

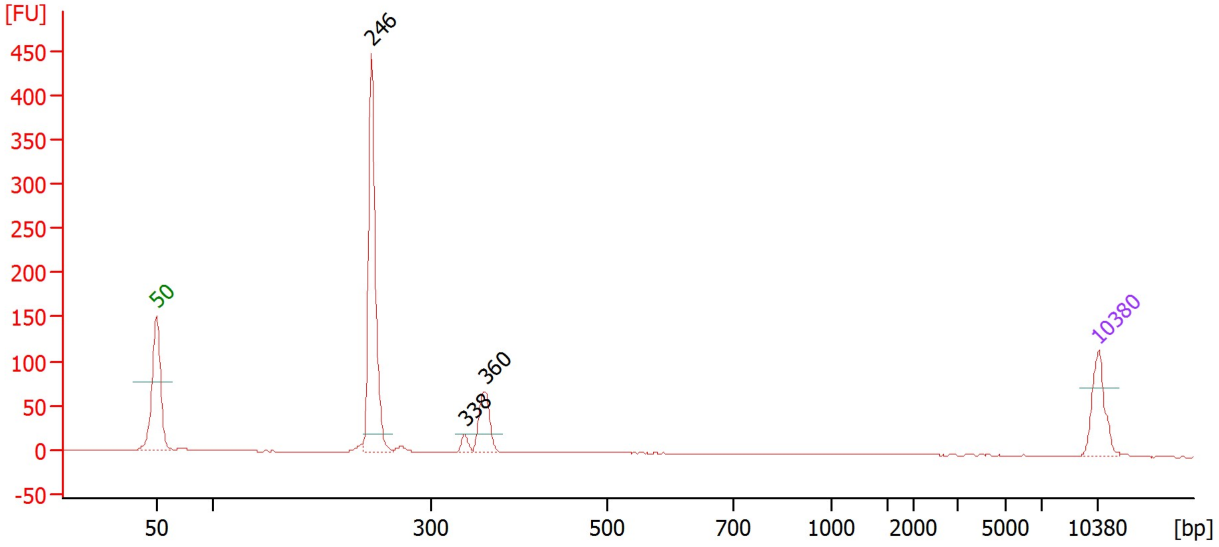

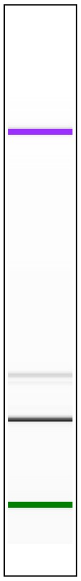

Overall Results for sample 3 : **Sample 3**

Number of peaks found: 3

Peak table for sample 3 : **Sample 3**

Peak		Size [bp]	Conc. [ng/µl]	Molarity [nmol/l]	Observations
1	◀	50	8.30	251.5	Lower Marker
2		246	15.51	95.7	
3		338	0.63	2.8	
4		360	2.95	12.4	
5	▶	10,380	4.20	0.6	Upper Marker

 © Copyright 2003 - 2009 Agilent Technologies, Inc.

Assay Class: DNA 7500 Created: 10/6/2021 4:45:38 PM
Data Path: C:\...ety\2100 expert_DNA 7500_DE13805338_2021-10-06_16-45-39.xad Modified: 10/6/2021 5:20:32 PM

Electropherogram Summary Continued ...

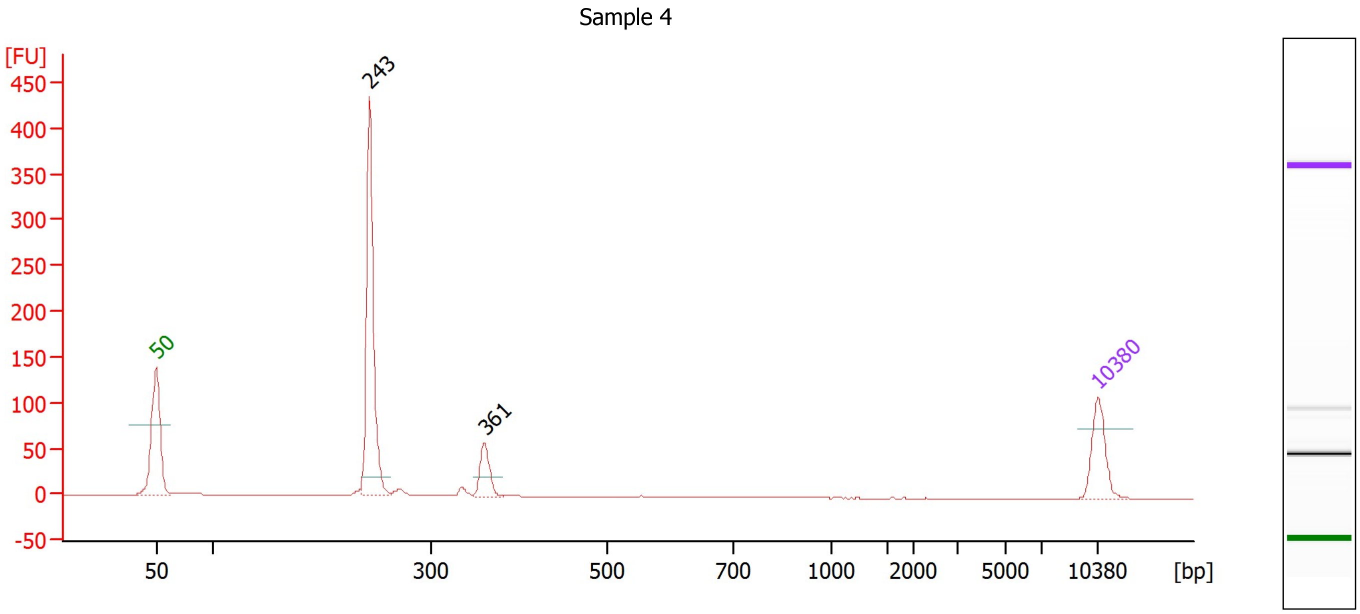

Overall Results for sample 4 : Sample 4

Number of peaks found: 2

Peak table for sample 4 : Sample 4

Peak		Size [bp]	Conc. [ng/µl]	Molarity [nmol/l]	Observations
1	◀	50	8.30	251.5	Lower Marker
2		243	15.04	93.7	
3		361	2.28	9.6	
4	▶	10,380	4.20	0.6	Upper Marker

 © Copyright 2003 - 2009 Agilent Technologies, Inc.

Assay Class: DNA 7500 Created: 10/6/2021 4:45:38 PM
Data Path: C:\...ety\2100 expert_DNA 7500_DE13805338_2021-10-06_16-45-39.xad Modified: 10/6/2021 5:20:32 PM

Electropherogram Summary Continued ...

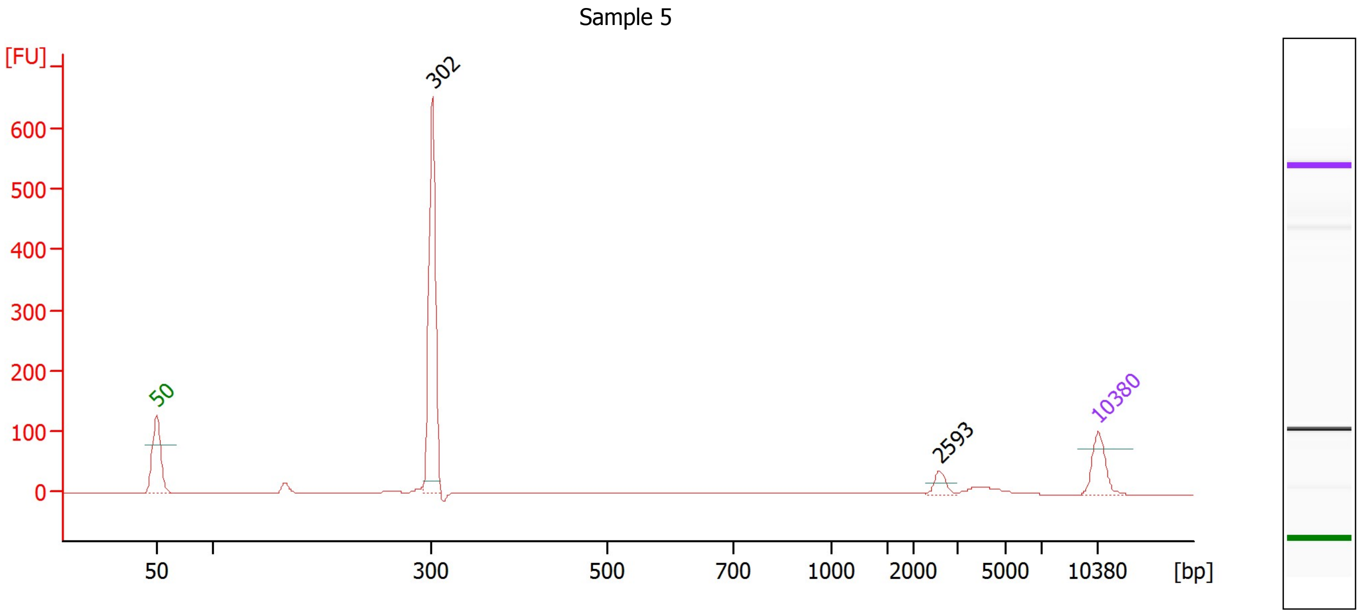

Overall Results for sample 5 : **Sample 5**

Number of peaks found: 2

Peak table for sample 5 : **Sample 5**

Peak		Size [bp]	Conc. [ng/µl]	Molarity [nmol/l]	Observations
1	◀	50	8.30	251.5	Lower Marker
2		302	21.16	106.3	
3		2,593	1.33	0.8	
4	▶	10,380	4.20	0.6	Upper Marker

 © Copyright 2003 - 2009 Agilent Technologies, Inc.

Assay Class: DNA 7500
Data Path: C:\...ety\2100 expert_DNA 7500_DE13805338_2021-10-06_16-45-39.xad

Created: 10/6/2021 4:45:38 PM
Modified: 10/6/2021 5:20:32 PM

Electropherogram Summary Continued ...

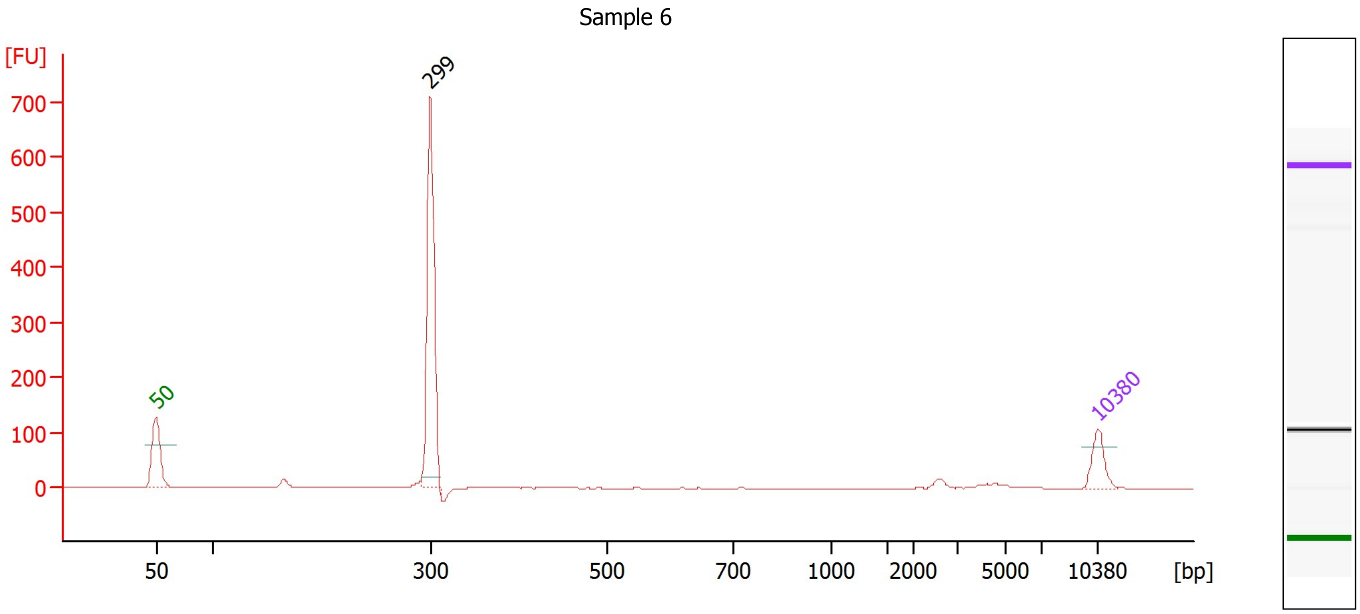

Overall Results for sample 6 : **Sample 6**

Number of peaks found: 1

Peak table for sample 6 : **Sample 6**

Peak		Size [bp]	Conc. [ng/µl]	Molarity [nmol/l]	Observations
1	◀	50	8.30	251.5	Lower Marker
2		299	25.73	130.4	
3	▶	10,380	4.20	0.6	Upper Marker

 © Copyright 2003 - 2009 Agilent Technologies, Inc.

Assay Class:	DNA 7500	Created:	10/6/2021 4:45:38 PM
Data Path:	C:\...ety\2100 expert_DNA 7500_DE13805338_2021-10-06_16-45-39.xad	Modified:	10/6/2021 5:20:32 PM

Electropherogram Summary Continued ...

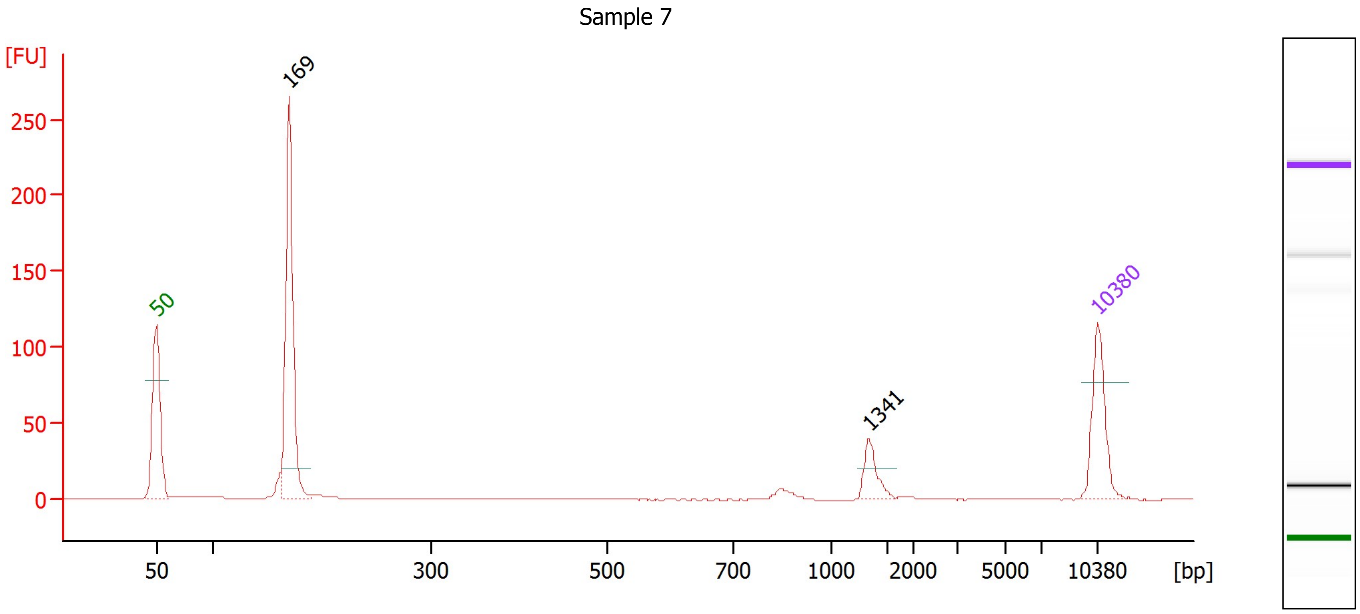

Overall Results for sample 7 : **Sample 7**

Number of peaks found: 2

Peak table for sample 7 : **Sample 7**

Peak		Size [bp]	Conc. [ng/µl]	Molarity [nmol/l]	Observations
1	◀	50	8.30	251.5	Lower Marker
2		169	10.57	94.9	
3		1,341	1.51	1.7	
4	▶	10,380	4.20	0.6	Upper Marker

 © Copyright 2003 - 2009 Agilent Technologies, Inc.

Assay Class: DNA 7500
Data Path: C:\...ety\2100 expert_DNA 7500_DE13805338_2021-10-06_16-45-39.xad
Created: 10/6/2021 4:45:38 PM
Modified: 10/6/2021 5:20:32 PM

Electropherogram Summary Continued ...

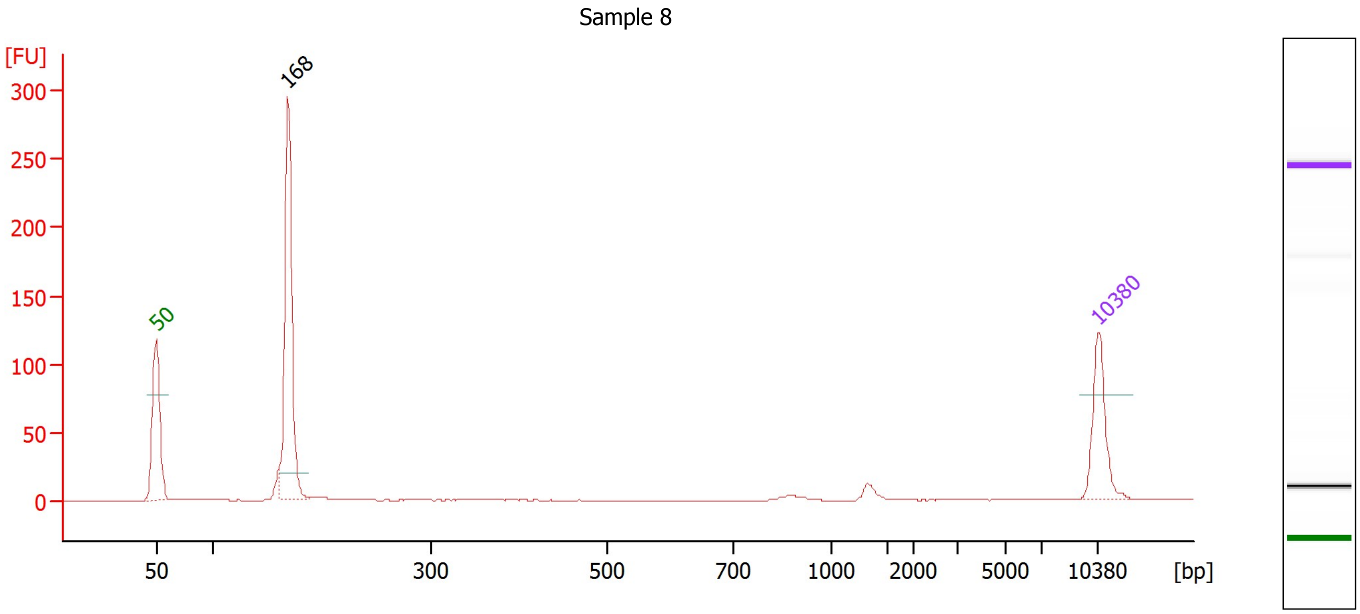

Overall Results for sample 8 : **Sample 8**

Number of peaks found: 1

Peak table for sample 8 : **Sample 8**

Peak		Size [bp]	Conc. [ng/µl]	Molarity [nmol/l]	Observations
1	◀	50	8.30	251.5	Lower Marker
2		168	11.15	100.3	
3	▶	10,380	4.20	0.6	Upper Marker

 © Copyright 2003 - 2009 Agilent Technologies, Inc.

Assay Class: DNA 7500 Created: 10/6/2021 4:45:38 PM
Data Path: C:\...ety\2100 expert_DNA 7500_DE13805338_2021-10-06_16-45-39.xad Modified: 10/6/2021 5:20:32 PM

Gel Image

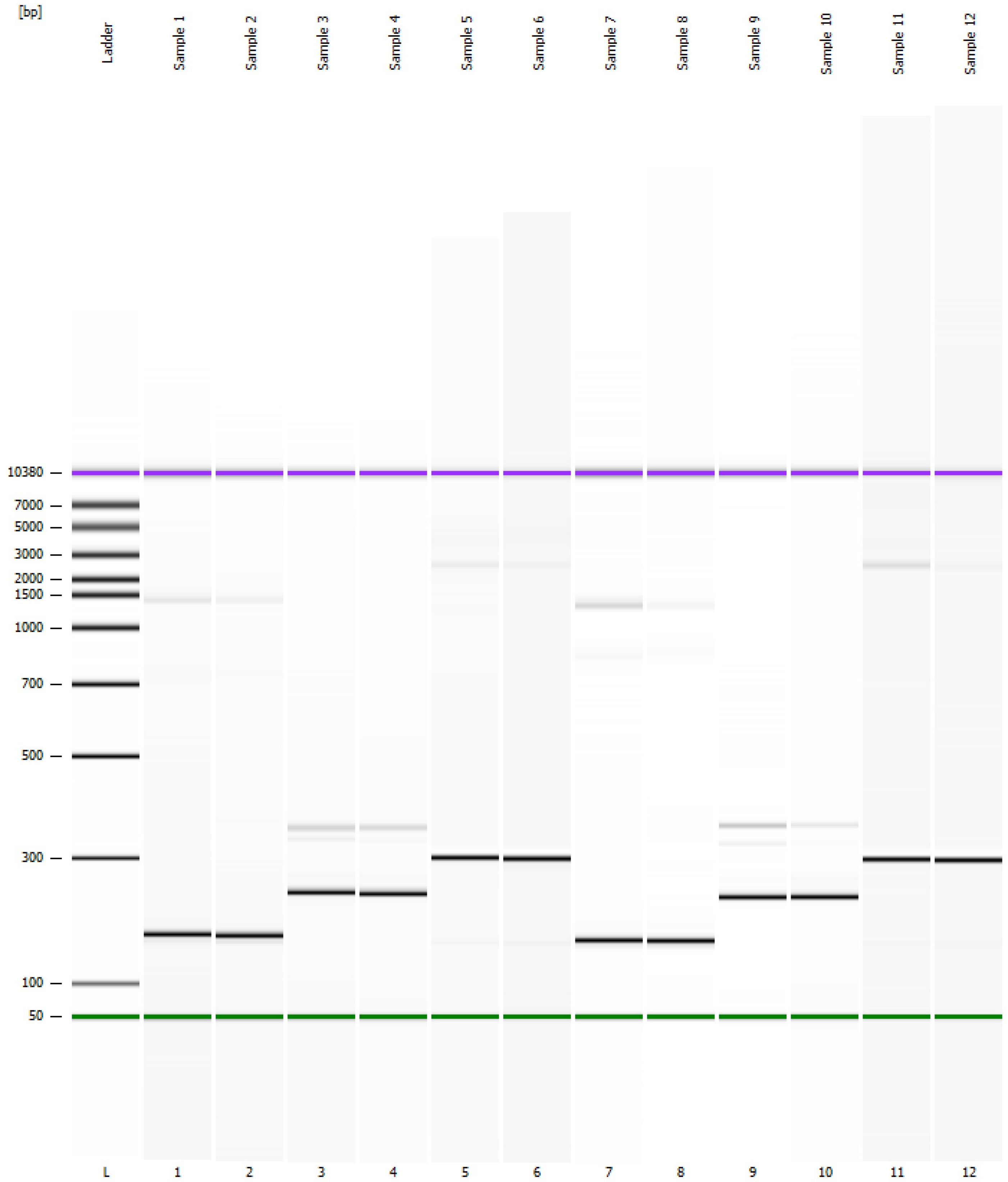

 © Copyright 2003 - 2009 Agilent Technologies, Inc.
