# Supplementary material for: Interleukin-2-mediated NF-κB-dependent mRNA splicing modulates interferon gamma protein production
Source: EMBO Rep. 2024 Nov 22;26(1):16–35. doi: 10.1038/s44319-024-00324-1 (PMC11724048; doi:10.1038/s44319-024-00324-1)
Supplement: Supplementary file 3 — Source data Fig. 2 [file 44319_2024_324_MOESM3_ESM.zip › 2B/2b original gels for bioanalyzer data.pdf]

NK2 Repeat 1

3hr treatment nucleus

6 hr treatment nucleus

IL12	+	+	+	+	+	+	+	+	+	+	+	+
IL2	-	+	-	+	-	+	-	+	-	+	-	+

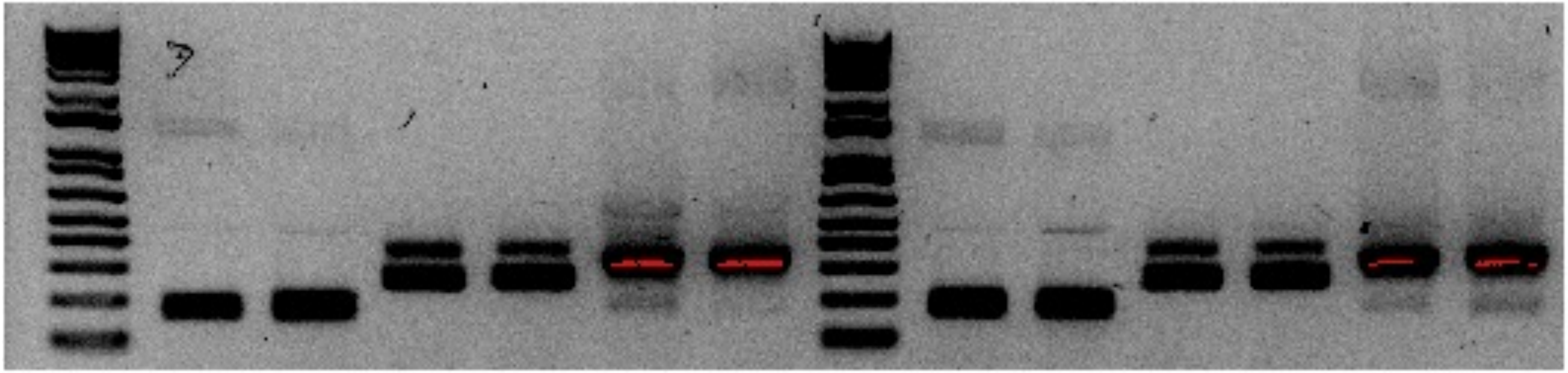

Exon 1- Ex2 Ex 2-Ex3 Ex 3- Ex4

Exon 1- Ex2 Ex 2-Ex3 Ex 3- Ex4

NK2 Repeat 2

1hr treatment nucleus

3hr treatment nucleus

6 hr treatment nucleus

IL12 + + + + + + IL12 + + + + + + IL12 + + + + + +

IL2 - + - + - + IL2 - + - + - + IL2 - + - + - +

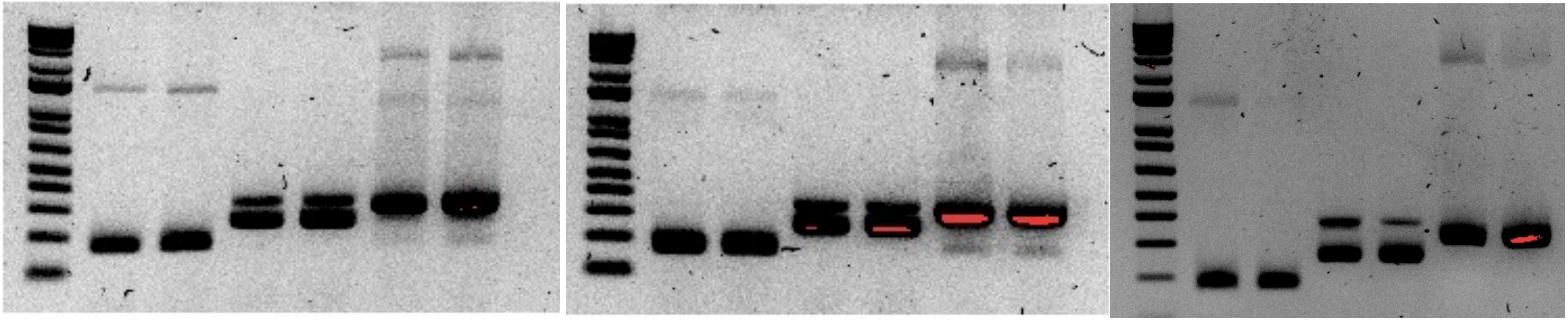

Exon 1- Ex2 Ex 2-Ex3 Ex 3- Ex4 Exon 1- Ex2 Ex 2-Ex3 Ex 3- Ex4 Ex 1- Ex2 Ex 2-Ex3 Ex 3- Ex4

NK2 Repeat 3

1hr treatment nucleus

IL12 + + + + + +

IL2 - + - + - +

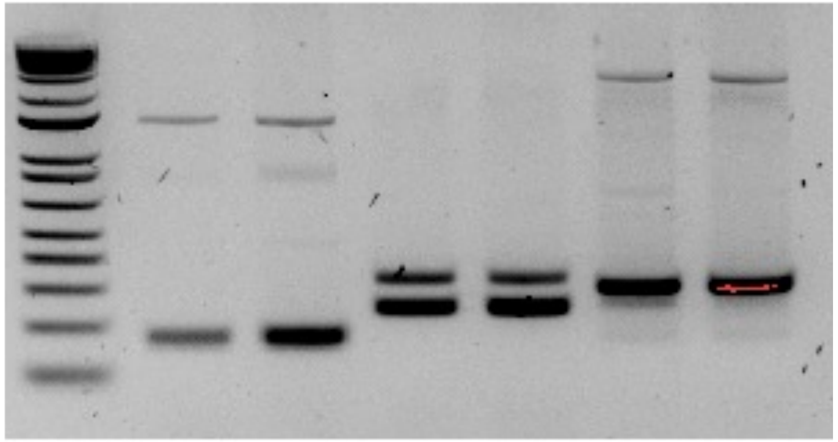

Exon 1- Ex2 Ex 2-Ex3 Ex 3- Ex4

3hr treatment nucleus

IL12 + + + + + +

IL2 - + - + - +

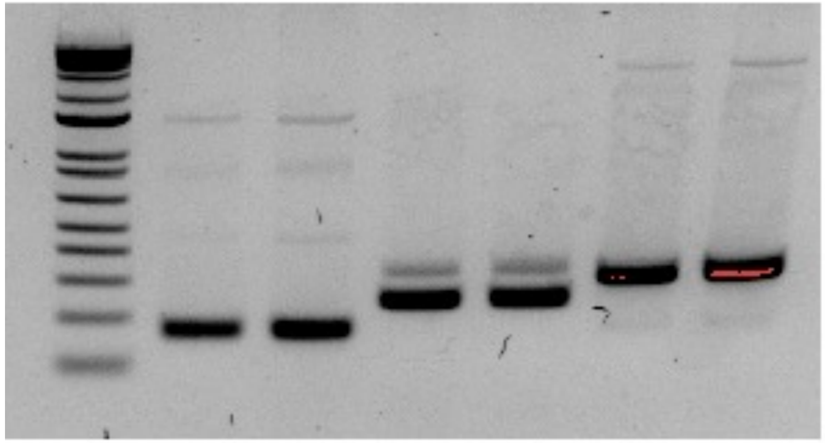

Exon 1- Ex2 Ex 2-Ex3 Ex 3- Ex4

6 hr treatment nucleus

IL12 + + + + + +

IL2 + - + - + -

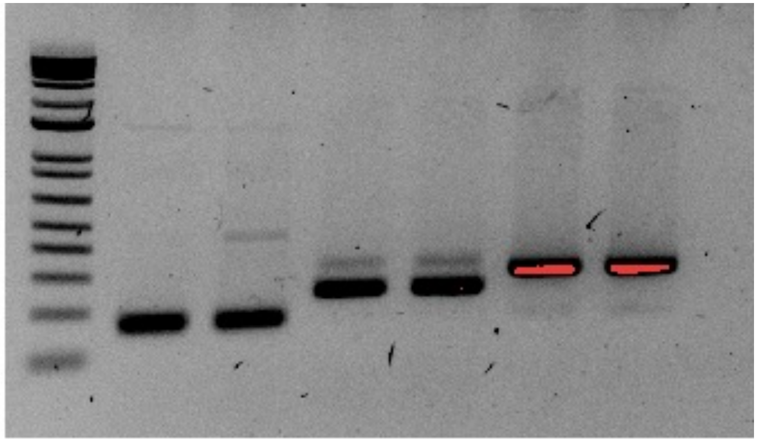

Ex 1- Ex2 Ex 2-Ex3 Ex 3- Ex4
