# Supplementary figures and images for: Interleukin-2-mediated NF-κB-dependent mRNA splicing modulates interferon gamma protein production
Source: EMBO Rep. 2024 Nov 22;26(1):16–35. doi: 10.1038/s44319-024-00324-1 (PMC11724048; doi:10.1038/s44319-024-00324-1)

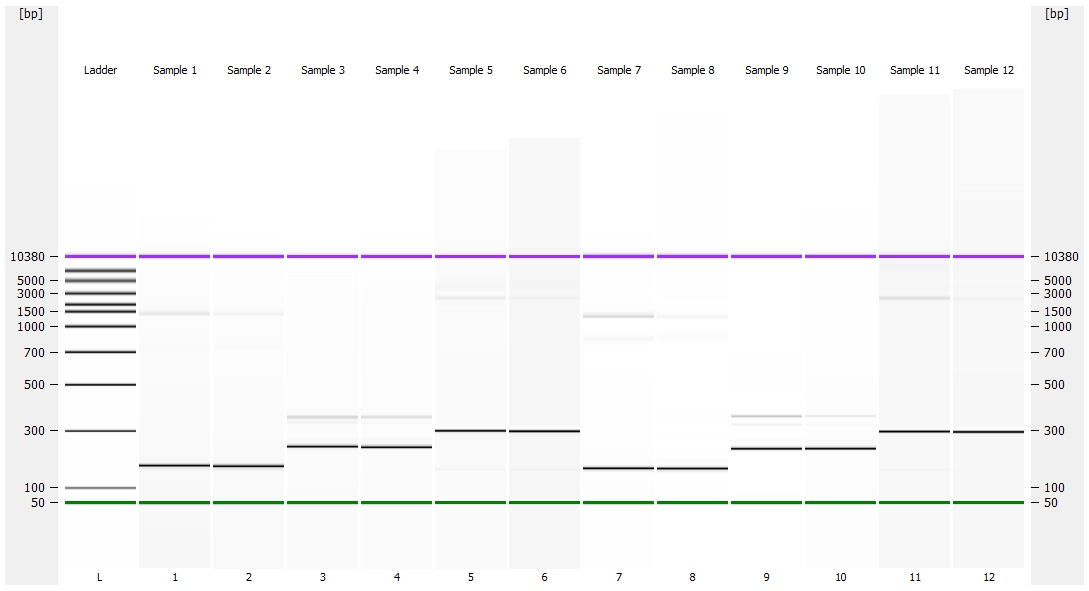

Supplement: Supplementary file 3 — Source data Fig. 2 [file 44319_2024_324_MOESM3_ESM.zip › 2B/gel image 2100 expert_DNA 7500_DE13805338_2021-10-06_16-45-39_GEL.jpg]
