# Supplementary material for: Interleukin-2-mediated NF-κB-dependent mRNA splicing modulates interferon gamma protein production
Source: EMBO Rep. 2024 Nov 22;26(1):16–35. doi: 10.1038/s44319-024-00324-1 (PMC11724048; doi:10.1038/s44319-024-00324-1)
Supplement: Supplementary file 6 — Expanded View Figures [file 44319_2024_324_MOESM6_ESM.pdf]

Expanded View Figures

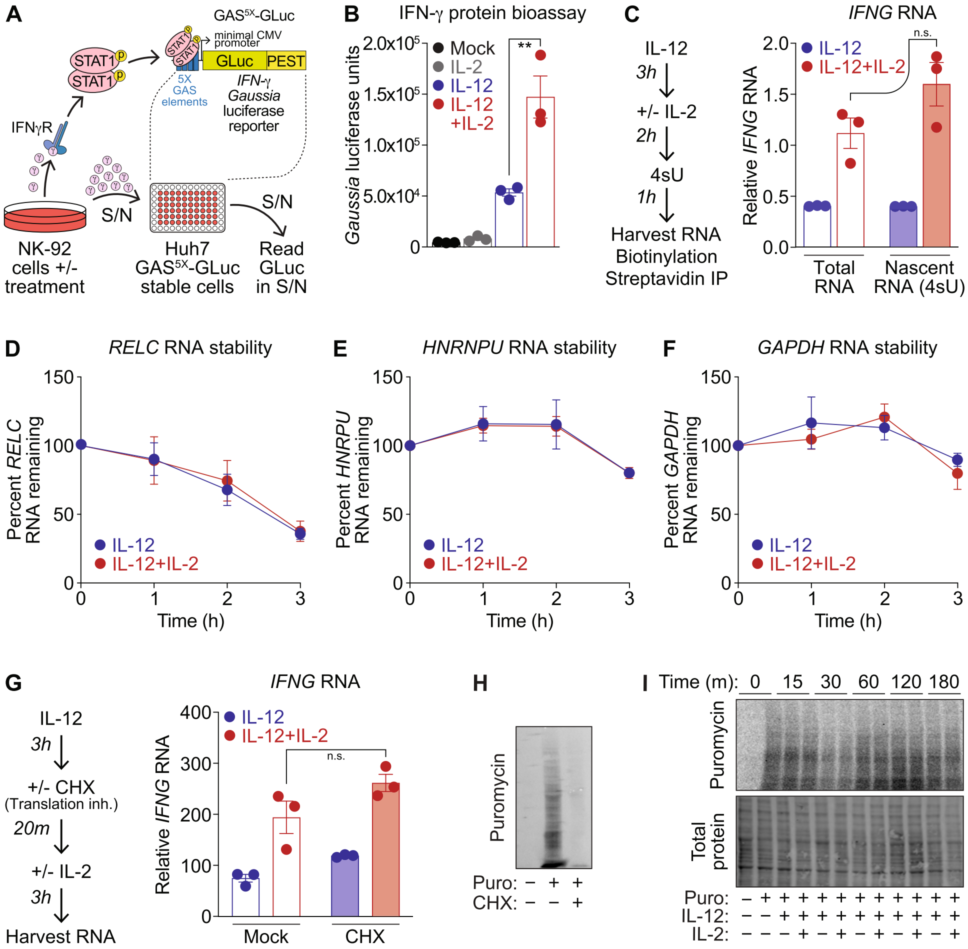

Figure EV1. IL-2 stimulation does not globally affect transcript stability or protein translation in NK92 cells.

(**A**) Schematic of IFNγ bioassay workflow for quantification of IFNγ in supernatants of treated NK92 cells via Huh7 *Gaussia* luciferase IRF1 GAS(5x) reporter cells. (**B**) Bioassay quantification of IFNγ protein production using supernatants from NK cells stimulated 24 h with IL-2 (100 u/mL), IL-12 (10 ng/mL) or IL-12 + IL-2 (IL-12 versus IL-12 + IL-2 stimulation, $p = 0.0037$). (**C**) qPCR analysis of 4SU labeled *IFNG* transcripts compared with total (4SU labeled + unlabeled) *IFNG* induction during IL-12 versus IL-12 + IL-2 treatment; normalized to *HPRT1*. Time course of (**D**) *RELC*, (**E**) *HNRNPU*, and (**F**) *GAPDH* mRNA stability in absence of nascent transcription during IL-2 stimulation, normalized to *RELC, HNRNPU*, and *GAPDH* levels at 3 h IL-12 treatment before addition of ActD, respectively. (**G**) qPCR analysis of *IFNG* mRNA induction during IL-2 stimulation in presence and absence of nascent translation, using cycloheximide (CHX) treatment (100 μg/mL) to halt protein synthesis. Normalized to *HPRT1* expression (**H**) Immunoblot depicting puromycin incorporation for cycloheximide protein synthesis halt control. (**I**) Immunoblot depicting puromycin incorporation in NK92 cells in NK92 cells stimulated over a time course of 0–3 h with IL-12 or IL-12 + IL-2. Total protein stain used for relative comparison of puromycin incorporation. Data information: Data in (**B–G**) are mean ± SEM of 3 biological replicates, (**B**) one-way ANOVA with Turkey's comparison test for multiple comparisons, (**C**) and (**G**) ratio paired T test with Holm-Šídák method for multiple corrections, (**H**) is representative of 3 biological replicates, (**I**) is representative of 2 biological replicates. $^{**}p \le 0.01$, n.s. is not significant.

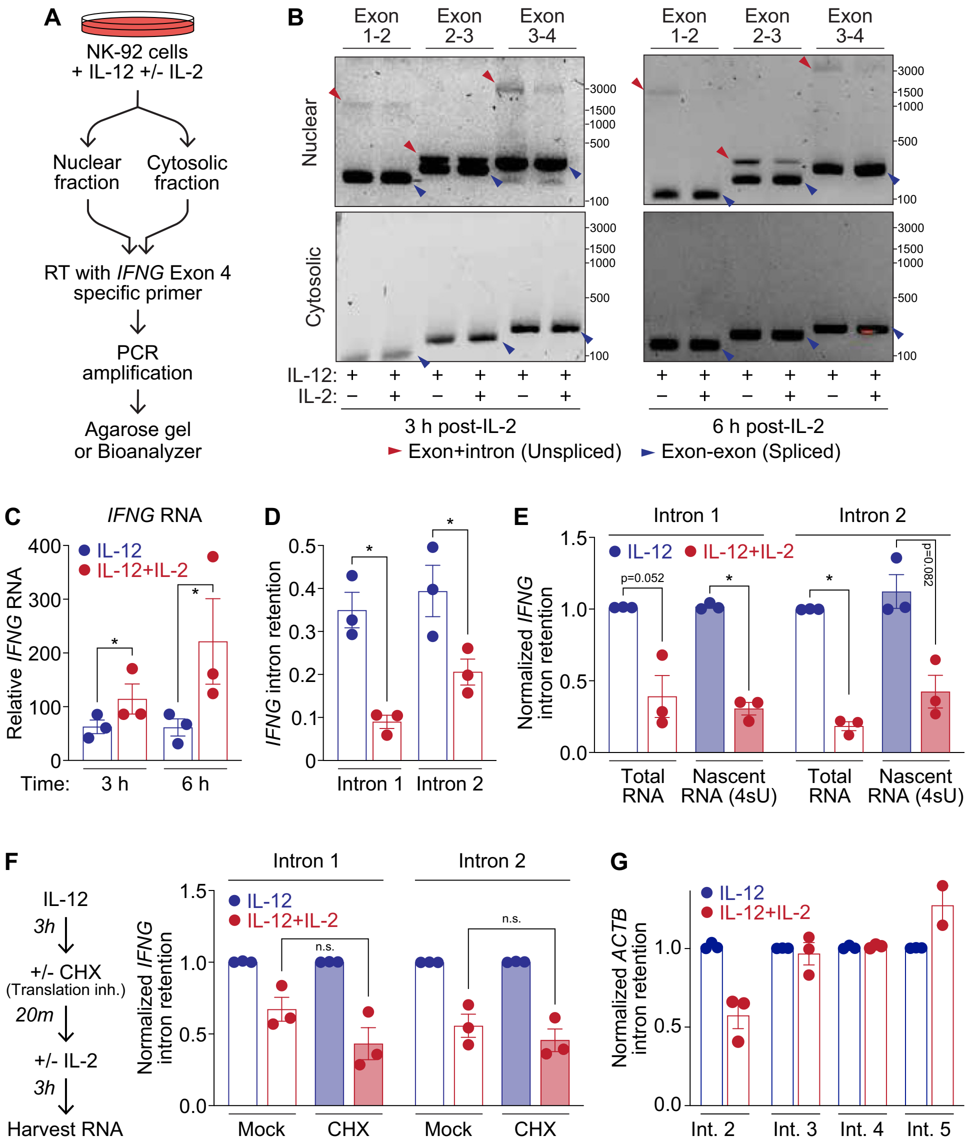

Figure EV2. IL-2 mediates *IFNG* intronic splicing during nascent translation and upon cycloheximide treatment.

(**A**) Schematic of NK92 stimulation and processing for determining *IFNG* intron retention via PCR as seen in Fig. 2B, EV1B. (**B**) Image of gels depicting PCR products from amplification of intra-exonal regions of *IFNG* from nuclear and cytosol fractionated NK92 cells stimulated with IL-12 or IL-12 + IL-2 for 3 or 6 h. Blue arrows depict fully spliced products while red arrows depict amplified intronic regions. (**C**) total spliced *IFNG* induction (IL-12 versus IL-12 + IL-2 stimulation, 3 h treatment $p = 0.04$; 6 h, $p = 0.044$) normalized to *HPRT1* expression during stimulations outlined in Fig. 2D. (**D**) SYBR qPCR analysis of *IFNG* intron retention in whole cell lysates during stimulation with IL-12 for 3 h with or without subsequent 3 h IL-2 stimulation. Each stimulation condition normalized to mock treatment condition. Intron expression normalized to *IFNG* 5′UTR, representing total mature plus unspliced *IFNG* mRNA as quantified by amplification of the region spanning the 5′UTR into the coding region of Exon 1 as control (Intron 1, IL-12 versus IL-12 + IL-2 stimulation, $p = 0.014$; Intron 2, $p = 0.025$). (**E**) SYBR qPCR analysis of *IFNG* intron retention in nascently transcribed or total mRNA from whole cell lysates stimulated for 6 h with IL-12 or IL-12 + IL-2. Cells were pulsed with 4SU between 5- and 6-h stimulation. Intron expression normalized to *IFNG* 5′UTR (Intron 1: 4SU treatment, IL-12 versus IL-12 + IL-2, $p = 0.013$; Intron 2: Mock, IL-12 versus IL-12 + IL-2 stimulation, $p = 0.02$). (**F**) SYBR qPCR analysis of *IFNG* intron retention upon IL-2 treatment with and without translation inhibition via CHX (100 µg/mL) treatment. Normalized to total mature plus unspliced *IFNG* expression via 5′UTR-Exon 1 amplification. (**G**) Analysis of intron retention in *ACTB* during IL-12 or IL-12 + IL-2 stimulation for 6 h, normalized to total expression of *ACTB* via amplification of Exon 6/3′UTR. Data information: Data in (**B**) representative of 3 biological replicates. Data in (**C**–**G**) depict mean ± SEM of 3 biological replicates, where (**C**) is analyzed by ratio paired T test and (**D**–**F**) paired T test with Holm-Šídák method for multiple corrections *$p \leq 0.05$, n.s. is not significant.

 © The Author(s)

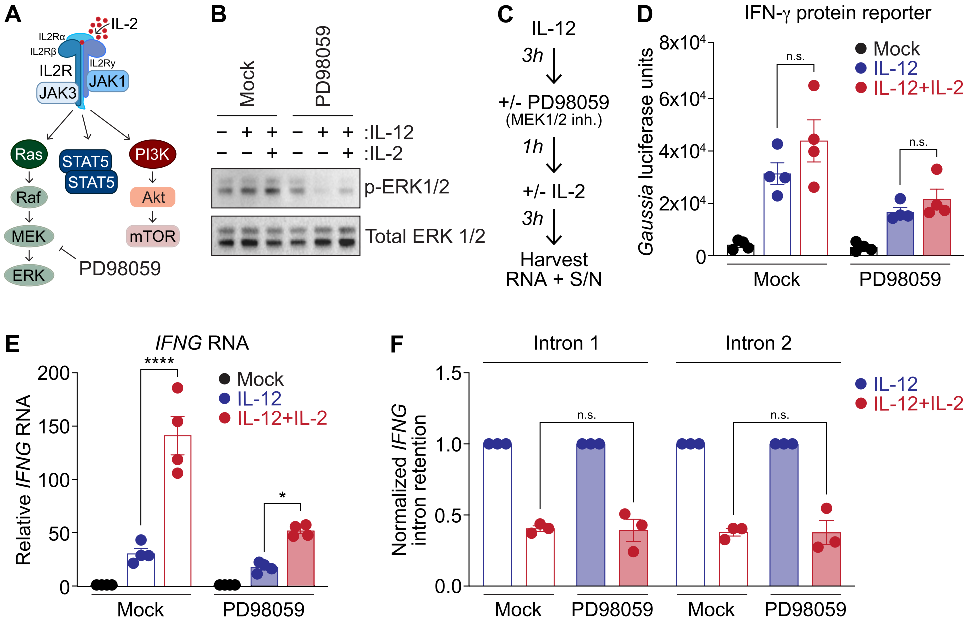

Figure EV3. ERK signaling does not post-transcriptionally regulate *IFNG* mRNA processing.

(**A**) Schematic of canonical IL-2R signaling. (**B**) Immunoblot confirming effect of inhibitor PD98059 (10 μM) on preventing phosphorylation ERK1/2 downstream of MEK1/2 inhibition during IL-12 and IL-2 treatment in NK92 cells (**C**) schematic of PD98059 treatment in determining effect on IFNγ induction and splicing. (**D**) *Gaussia* luciferase bioassay for IFNγ protein quantification (**E**) total *IFNG* transcript induction and (**F**) *IFNG* intron retention upon treating cells with PD98059 (25 μM) prior to IL-2 treatment, normalized to total spliced plus unspliced *IFNG* (Mock treatment, IL-12 versus IL-12 $p < 0.0001$; PD98059 treatment, IL-12 versus IL-12 + IL-2 $p = 0.017$). Data information: Data in (**D**–**F**) is mean ± SEM for 3 or 4 biological replicates. (**B**) is representative of 3 biological replicates. (**D**–**E**) analyzed two-way ANOVA with Turkey's test for multiple comparisons and (**F**) by paired T test with Holm-Šídák method for multiple corrections. *$p \leq 0.05$, ****$p \leq 0.0001$, n.s. is not significant.

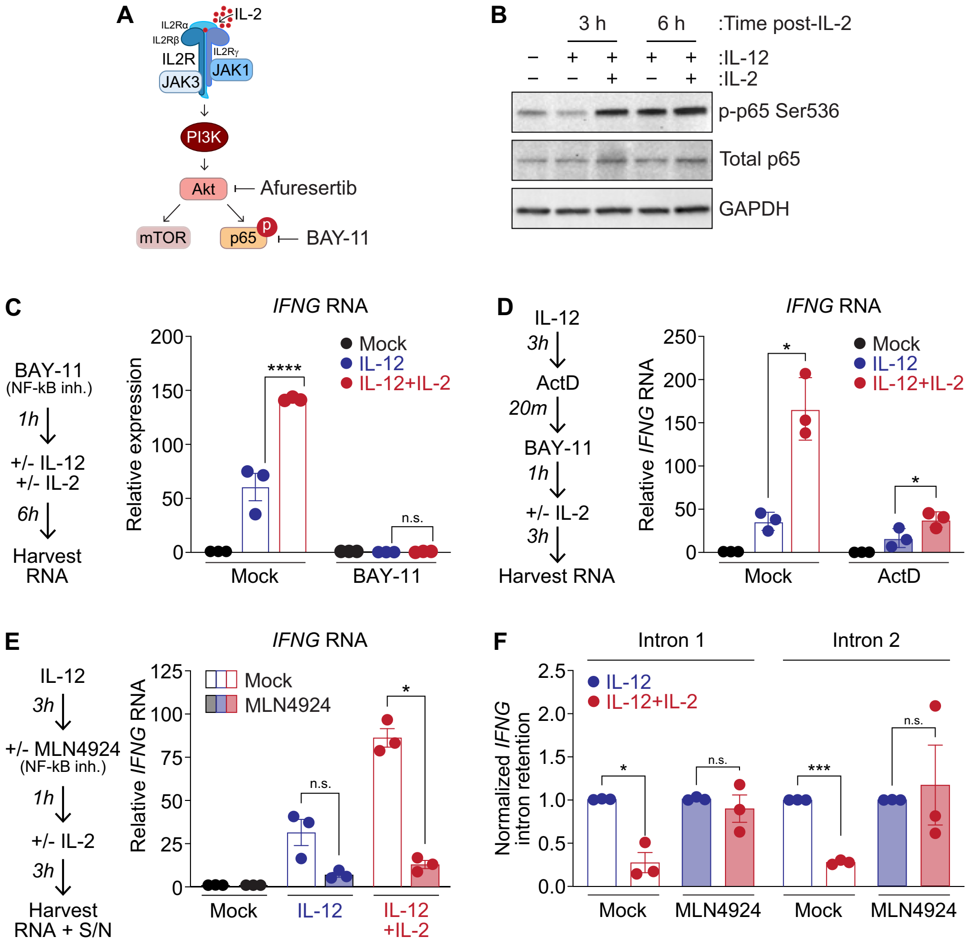

Figure EV4. NF-κB signaling downstream of IL-2 is required for post-transcriptional regulation of *IFNG* mRNA.

(**A**) Schematic depicting alternative signaling through Akt downstream of the IL-2 receptor in NK cells (**B**) immunoblot for total and phosphorylated NF-κB p65 NK cells treated with IL-12 or IL-12 + IL-2 for 3 or 6 h with GAPDH as loading control (**C**) qPCR analysis of *IFNG* induction upon pre-treatment with the NF-κB inhibitor BAY-11, prior to any stimulation (Mock treatment, IL-12 versus IL-12 + IL-2 comparison $p < 0.0001$). (**D**) *IFNG* mRNA induction normalized to *HPRT1* with all samples normalized to the no stimulation condition within the mock treatment to confirm functioning ActD halt of nascent transcription (Mock treatment, IL-12 versus IL-12 + IL-2, $p = 0.0397$; ActD treatment, IL-12 versus IL-12 + IL-2, $p = 0.0257$). (**E**) qPCR analysis of total *IFNG* mRNA induction (Mock versus MLN4924 treatment during IL-12 + IL-2 stimulation, $p = 0.015$) and (**F**) SYBR qPCR analysis of *IFNG* mRNA intron retention upon inhibiting NF-κB signaling with MLN4924 (10 μM), (Intron 1, Mock treatment, IL-12 versus IL-12 + IL-2 $p = 0.049$; Intron 2 Mock treatment $p = 0.0004$). Data information: (**B**) is representative of 5 biological replicates where 4 of 5 showed the depicted result. Data in (**C–F**) is mean ± SEM for 3 biological replicates with (**C**) analyzed by one-way ANOVA with Turkey's test for multiple comparisons, (**D**) analyzed by two-way ANOVA with Turkey's test for multiple comparisons, (**E**) analyzed by ratio paired T test and (**F**) by paired T test with Holm-Šídák method for multiple corrections *$p \le 0.05$, ***$p \le 0.001$, ****$p \le 0.0001$, n.s. is not significant.

 © The Author(s)
